# Nanoscale Technologies in Highly Sensitive Diagnosis of Cardiovascular Diseases

**DOI:** 10.3389/fbioe.2020.00531

**Published:** 2020-06-05

**Authors:** Chaohong Shi, Haotian Xie, Yifan Ma, Zhaogang Yang, Jingjing Zhang

**Affiliations:** ^1^Department of Rehabilitation Medicine, The First People’s Hospital of Wenling, Wenzhou Medical University, Wenling, China; ^2^Department of Mathematics, The Ohio State University, Columbus, OH, United States; ^3^Department of Chemical and Biomolecular Engineering, The Ohio State University, Columbus, OH, United States; ^4^Department of Radiation Oncology, University of Texas Southwestern Medical Center, Dallas, TX, United States

**Keywords:** cardiovascular disease (CVD), biomarker, molecular imaging, diagnostic, nanotechnology

## Abstract

Cardiovascular diseases (CVD) are the leading cause of death and morbidity in the world and are a major contributor to healthcare costs. Although enormous progress has been made in diagnosing CVD, there is an urgent need for more efficient early detection and the development of novel diagnostic tools. Currently, CVD diagnosis relies primarily on clinical symptoms based on molecular imaging (MOI) or biomarkers associated with CVDs. However, sensitivity, specificity, and accuracy of the assay are still challenging for early-stage CVDs. Nanomaterial platform has been identified as a promising candidate for improving the practical usage of diagnostic tools because of their unique physicochemical properties. In this review article, we introduced cardiac biomarkers and imaging techniques that are currently used for CVD diagnosis. We presented the applications of various nanotechnologies on diagnosis within cardiac immunoassays (CIAs) and molecular imaging. We also summarized and compared different cardiac immunoassays based on their sensitivities and working ranges of biomarkers.

## Introduction

Cardiovascular diseases (CVDs) are the most common causes of death in the world (Ho, 2018). CVDs can be medically defined as a group of disorders involving heart, brain, and blood vessels, including but not limited to coronary heart diseases, peripheral arterial diseases, rheumatic heart diseases, deep vein thrombosis, and cerebrovascular diseases – all of which result in ischemia and tissue death (Yang et al., 2009, 2012; Laflamme et al., 2012; Chakrabarti et al., 2013; Yu et al., 2015; Fan et al., 2020a, b). General CVDs can be characterized into five categories: atherosclerosis, acute myocardial infarction (AMI), heart failure (HF), stroke, and hypertension (Lichtenstein and Matthan, 2007; Govindappa et al., 2020; Joshi et al., 2020). Individuals who demonstrate tobacco smoking, high levels of low-density lipoproteins (LDL)-associated cholesterol, glucose, and diabetes as well as overweight and obesity, are especially susceptible to CVD morbidity and mortality (D’Agostino et al., 2008). Effectively diagnosing individuals who are most susceptible to CVDs opens the door to optimal treatment, thereby lowering the death rate. Given that early-stage CVDs demonstrate a high survival rate, predicting CVDs early on is essential.

Current common clinical CVD diagnosis methods include electrocardiography (ECG), plain X-ray, computed tomography (CT), and magnetic resonance imaging (MRI), and other MOI techniques (Anderson et al., 2013). ECG measures variations in the conduction system of the heart and monitors chest pain in AMI patients (Fesmire et al., 1998). CT scans X-ray images around the body and generates slices images of bones, blood vessels and tissues, which is appropriate for CVD diagnosis on grounds of its high signal contrast and accuracy (Kirkpatrick et al., 2003). MRI has been widely used in atherosclerosis and stroke detection given it scans three-dimensional images of bodies in a non-invasive manner (Pykett et al., 1983). However, these traditional methods were limited to low sensitivity and specificity.

To overcome these aforementioned difficulties, various new platforms such as cardiac immunoassays (CIAs) and advanced molecular imaging (MOI) were introduced, which significantly improved the efficiency of CVD diagnosis over the past decades (Qureshi et al., 2012; Osborn and Jaffer, 2013). Cardiac biomarkers are substances in the blood when the heart and brain are damaged or act abnormally. For example, cardiac troponin I (cTnI) has been demonstrated as a promising biomarker for AMI (Apple et al., 1997). MOI is capable of identifying cellular and molecular biology process, however, each technique has advantages and limitations. Therefore, advanced MOI combined different MOI techniques have been invented (e.g., dual-module, triple-module-CT) to obtain more detailed imaging information, which has increased the accuracy of diagnostic results (Hur et al., 2011, 2012).

Despite the great merits of previous methods, early-stage diagnosis is still challenging due to its complex pathophysiology, vague symptoms, and low expression levels of cardiac biomarkers. These difficulties increase the aggravation and mortality of CVDs. For instance, atherosclerosis shows no signs or symptoms and extremely low-level of related biomarkers in some patients even after a heart attack (Libby, 2002). Moreover, quick and convenient measurements are inadequate in addressing the expanded needs of CVD patients. Hence, rapid, accurate, and highly sensitive and specific platforms are needed for early-stage CVDs.

**FIGURE 1:**
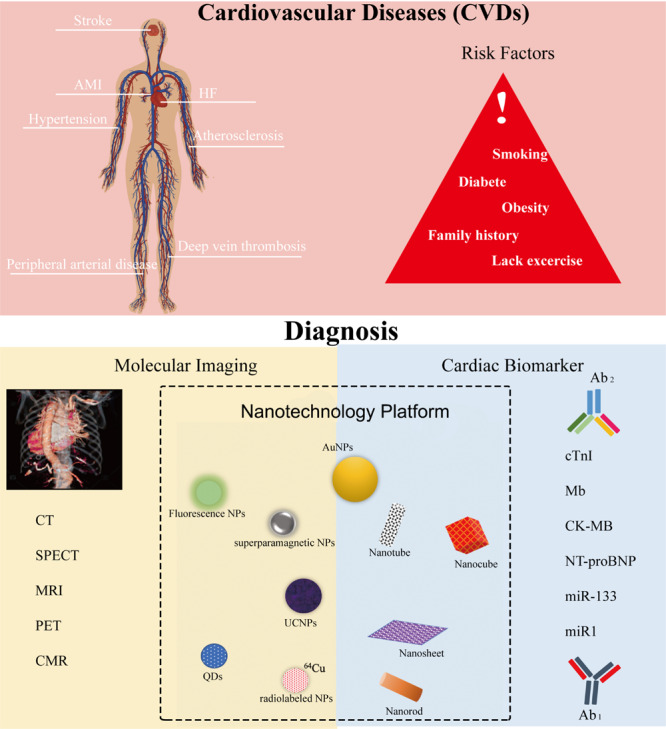
Cardiovascular diseases (CVDs), their risk factors, and their diagnosis settings include molecular imaging and cardiac immunoassay within the nanotechnology platform. Ab_1_, capture antibodies that immobilize the targets. Ab_2_, detection or secondary antibodies that detect and quantify the targets.

Nanotechnology involves nanoscale dimension systems (Johnson, 2012; Zhou et al., 2014), has specific physicochemical properties that make them appealing for improving current diagnosis (Kuriyama et al., 2011; Sun et al., 2016, 2019; Chen Z. et al., 2017; Liu et al., 2017; Yang et al., 2020a). Nanomaterials have been extensively applied to CIAs, including electrochemiluminescence (ECL), Electrochemical (EC), and photoelectrochemistry (PEC) due to their unique optical property, electrical property, and excellent biocompatibility (Figure 1) (Abdorahim et al., 2016). For example, Gold nanoparticles (AuNPs) can be incorporated with biotinylated antibodies to reduce non-specific binding, or conjugated with biomolecules with specific physical properties [e.g., hybridization chain reaction (HCR)] for signal amplification. Liu G. et al. (2016) detected cTnI via antigen-antibody affinity with a low limit of detection using AuNPs and graphene oxide. AuNPs and other metal nanoparticles can modify substrates (e.g., boron nitride nanosheets, titanium) in EC assays as well because of their attractive electrical properties (Golberg et al., 2010). Other nanomaterials, like silica/Pt NPs, are common signal enhancers in PEC and surface plasmon resonance (SPR) thanks to their efficient photocurrent quenching ability and unique plasmonic properties (Homola et al., 1999). Besides, upconversion nanoparticles (UCNPs) have been used in fluorescence assay because of their excellent photon conversion ability (Haase and Schäfer, 2011). Also, knotted and hollow nanomaterials (e.g., nanosheets, nanotubes, nanowires, and nanoclusters) with the large surface area that increases loading efficiency of biomolecules for signal enhancement (Kong et al., 2000). Nanomaterials also play important roles in MOI. Nanomaterials coupled with photoacoustic, fluorescent, radioactive, paramagnetic substances can work as contrast agents in MOI settings to enhance their detection signal (Van Schooneveld et al., 2010).

In this review, we will introduce the recent progress of nanotechnology for diagnosing CVDs. The nanotechnology facilitated CIA and MOI applications will be covered. Firstly, we summarized common clinical cardiac biomarkers and MOI settings. Then the applications on nanotechnology to identify cardiac biomarkers within different platforms based on unique properties of various nanomaterials will be introduced, including ECL, fluorescence, PEC, EC Surface-enhanced Raman scattering (SERS), SPR, Field effect transistor (FET), Enzyme-Linked Immunosorbent Assay (ELISA), and lateral flow assay (LFA). We compared different CIAs based on their sensitivities and working ranges of cardiac biomarkers as well. Besides, we concluded current diagnosis achievements on MOI with the help of functionalized nanoparticle.

## CVD Diagnostic Settings

### Cardiac Immunoassay (CIA)

Cardiac biomarkers present in human bodyfluids are reliable and reproducible indicators of the risk and progression of CVDs. They can be detected and quantified through various CIAs based on antigen-antibody immunoaffinity. Measuring expression levels of cardiac biomarkers within CIAs shows advantages, including high sensitivity, rapid, cheap, and non-invasive for the prediction and diagnosis of disease. Cardiac biomarkers can be classified as circulating biomarkers and exosomal biomarkers. Circulating biomarkers present in bodyfluids freely, and exosomal biomarkers are bound into or on the surface of extracellular vesicles (EVs) that are mobile and secreted from cells.

#### Cardiac Circulating Biomarkers

Circulating biomarkers include miRNA, mRNA, long-non-coding RNA, proteins, and other substances that present in human blood, milk, saliva, urine, and cerebral spinal biofluids (Durrani-Kolarik et al., 2017; Wang et al., 2017a, b; Wang X. et al., 2018; Yang et al., 2020b). Currently, several cardiac biomarkers, such as cardiac troponin I (cTnI), troponin I (TnI), myoglobin (MB), C-reactive protein (CRP), and creatine kinase-MB (CK-MB), have attracted interests as potential biomarkers for AMI (Christenson and Christenson, 2013). Particularly, cTnI has high specificity and sensitivity toward AMI (Jo et al., 2015), and MB is a good candidate for early diagnosis of AMI (Korff et al., 2006). Myeloperoxidase (MPO), glycogen phosphorylase isoenzyme BB (GPBB), B-type natriuretic peptide (BNP), N-terminal pro-B-type natriuretic peptide (NT-proBNP), C-type natriuretic peptide (c-TNP), Matrix metalloproteinase-8 (MMP-8), MMP-9 and tissue inhibitor of MMP-8 (TIMP-1), and leukotriene B4 are promising biomarkers as well (Anderon, 2005).

Recently, researchers discovered that some miRNAs circulating in serum or plasma were closely correlated with CVDs. For example, miR-208 was undetectable in healthy donors but was detected successfully in 90.9% of AMI patients (Ji et al., 2009). There are almost 50 circulating miRNAs proven to be relevant to CVDs. Specifically, miR-208a, miR-208b, and miR133 are up-regulated AMI biomarkers, while miR150, let-7b, and miR-126 are down-regulation AMI biomarkers (Corsten et al., 2010; Wang et al., 2010; Gidlöf et al., 2011; Long et al., 2012a, b; Devaux et al., 2013; Friese et al., 2013; Li et al., 2013). Furthermore, miR-423, miR-18b, miR-499, miR-142, miR-320a, miR-22, miR-20b, and miR-26b are positively associated with HF, but miR-103 is shown to be low in HF patients (Corsten et al., 2010; Tijsen et al., 2010; Goren et al., 2012; Ellis et al., 2013; Marfella et al., 2013). Additionally, let-7e, Hcmv-miR-UL112, miR-605, miR623, miR-516b, miR-132 are found to be relatively high in plasma in hypertension cases. In contrast, miR-296, miR-133b, miR-625, miR-1236 are down-regulation biomarkers (Li et al., 2011). Moreover, miR-145, miR21 are up-regulating biomarkers for strokes, and miR-221, miR-210, miR-30a, and miR-126 are shown to be low in human blood in stroke cases (Zeng et al., 2011; Gan et al., 2012; Long et al., 2012b; Tsai et al., 2013). In summary, circulating miRNAs are relatively stable and serve as sufficiently sensitive biomarkers for CVD diagnosis.

#### Cardiac Exosomal Biomarkers

EVs are submicron-sized vesicles ranging from 30 to 1000 nm secreted by cells. EVs play important roles in transferring proteins, mRNA, miRNA, and other molecules among cells (Yang et al., 2016; Ma et al., 2019; Walters et al., 2019; Liu et al., 2019). Increasing evidence showed that exosomal miRNAs are involved in the pathogenesis of CVDs (Zamani et al., 2019). It has been observed that exosomal miRNAs mediate intercellular communication in cardiovascular systems and play an indispensable role in the control of cellular functions (Bang et al., 2014). As such, exosomal miRNAs can be used as biomarkers for diagnosing subjects with CVDs (Rehman et al., 2017).

Recently, researchers found miR-1, miR-133a, miR-21, and miR-499 are AMI-related biomarkers (Cheng et al., 2012; Oerlemans et al., 2012). Also, miR-192, miR-194, miR-34a, miR-423-5p, miR320a, miR-22, and miR-92b are highly associated with HF (Goren et al., 2012; Matsumoto et al., 2013). Besides, miR-223 isolated from serum EVs is a potential biomarker for stroke (Chen Y. et al., 2017). Additionally, exosomal membrane proteins or internal proteins, such as TNF-α and fibronectin are also potential CVD biomarkers (de Jong et al., 2012; Yu et al., 2012). Table 1 summarized the biomarkers mentioned above.

**TABLE 1:** Summary of cardiac biomarkers.

**Biomarker ID**	**Type**	**Pathology**	**Expression**	**References**
cTnI, TnI, MB, C-CRP, CK-MB, MPO, GPBB, NT-proBNP, c-TNP, MMP-8, MMP-9, TIMP-1, leukotriene B4, TNF-α, and fibronectin	Protein	AMI, HF, Hypertension, Stroke	Upregulated	Anderon, 2005; Korff et al., 2006; de Jong et al., 2012; Yu et al., 2012; Christenson and Christenson, 2013; Jo et al., 2015
miR-208, miR-208a, miR-208b, miR133, miR-1, miR-133a, miR-21, and miR-499	RNA	AMI	Upregulated	Ji et al., 2009; Corsten et al., 2010; Wang et al., 2010; Gidlöf et al., 2011; Cheng et al., 2012; Long et al., 2012a, b; Oerlemans et al., 2012; Devaux et al., 2013; Friese et al., 2013; Li et al., 2013
miR150, let-7b, and miR-126	RNA	AMI	Downregulated	Devaux et al., 2013; Friese et al., 2013; Li et al., 2013
miR-423, miR-18b, miR-499, miR-142, miR-320a, miR-22, miR-20b, miR-26b, miR-192, miR-194, miR-34a, miR-423-5p, miR320a, miR-22, and miR-92b	RNA	HF	Upregulated	Corsten et al., 2010; Tijsen et al., 2010; Goren et al., 2012; Ellis et al., 2013; Marfella et al., 2013; Matsumoto et al., 2013
miR-103	RNA	HF	Downregulated	Ellis et al., 2013
let-7e, Hcmv-miR-UL112, miR-605, miR623, miR-516b, miR-132	RNA	Hypertension	Upregulated	Li et al., 2011
miR-296, miR-133b, miR-625, miR-1236	RNA	Hypertension	Downregulated	Li et al., 2011
miR-145, miR21, miR-223	RNA	Stroke	Upregulated	Zeng et al., 2011; Gan et al., 2012; Long et al., 2012b; Tsai et al., 2013; Chen Y. et al., 2017
miR-221, miR-210, miR-30a, and miR-126	RNA	Stroke	Downregulated	Zeng et al., 2011; Long et al., 2012b

### Molecular Imaging

Molecular imaging (MOI) is a common diagnostic tool for CVD in clinical practice. When paired with other approaches, MOI can reveal individual biology, including blood vessels, the brain, and the heart (Jaffer et al., 2007). Common MOI approaches include photoacoustic tomography (PAT), MRI, CT, positron emission tomography (PET), and single photon emission computed tomography (SPECT). PAT utilizes the photoacoustic effect that converts optical adsorption into acoustic energy and generates high-resolution imaged under optically ballistic and diffusive modules (Xia et al., 2014). Various materials such as dyes, nanoparticles, and probes can work as functional contrasts in PAT for vascular imaging (Li and Wang, 2009). CT takes X-ray images from different angles around the human body and produces cross-sectional images of the body to reveal blood vessels, tissues and organs (Article, 2018). MRI generates anatomical images based on the changes of protons in the body with a strong magnetic field (Hashemi et al., 2012). MRI sensors first turn on the radiofrequency current that spins the protons out of equilibrium and then detects the time and amount of released energy of realigning randomized protons within the magnetic field after turning off the radiofrequency current (McRobbie et al., 2017). Detected parameters are translated into images for diagnosis. PET uses injected radioactive tracers to review and evaluate tissues. The radiotracers used in PET produce positrons after decaying. Positrons react with electrons and produce photons that aggregate in specific, disease-related areas of the human body (Shan et al., 2013). The combination of PET with CT or MRI can create detailed, specific images. Similar to PET, SPECT provides tomographic images by recording and translating the activities of radioactive tracers that have gamma ray emissions (Hutton, 2014).

## Nanotechnology-Based Cardiac Immunoassay

Various advanced techniques like ECL, PEC, SERS, SPR, and ELISA have been utilized to detect cardiac biomarkers with precision. Despite satisfactory results from previous methods, pursuing high sensitivity and accuracy in testing results is still an ongoing endeavor. Combining nanotechnologies with cardiac immunoassays may serve as a solution for early-stage CVD diagnosis. Nanotechnologies may reduce non-specific binding sites, provide high binding efficiency of cardiac targets, offer excellent signal amplification, and possess multiple functions. Table 2 summarized and compared performances of recent cardiac immunoassays based on various nanotechnology platforms.

**TABLE 2:** Comparison of cardiac biomarker detection in developed immunoassays.

**Target**	**Assay**	**Nanoplatform**	**Range**	**Detection limit**	**References**
cTnI	ECL	Au nanocluster	5 fg/mL to 50 ng/mL	1.01 fg/mL	Zhu et al., 2019
	ECL	Ag Nanoparticles	NA	0.58 fg/mL	Wang et al., 2019
	ECL	QDs	0.17–3 ng/mL	0.02 ng/mL	Lakshmanakumar et al., 2019
	Fluorescence	QDs	1 pg/mL to 10 ng/mL	0.227 pg/mL	Miao et al., 2019
	Fluorescence	Pd-Ir nanocubes	1 pg/mL to 1 ng/mL	0.31 pg/mL	Tan et al., 2019
	Fluorescence	AuNP	NA	0.019 ng/mL	Han and Kim, 2019
	PEC	QDs	0.5 pg/mL to 10 ng/mL	0.5 pg/mL	Xue et al., 2019
	PEC	Nanospheres and QDs	0.08 ng/mL to 40 ng/mL	0.026 ng/mL	Dong et al., 2020
	PEC	Mesoporous silica nanoparticles	1.2 fg/mL to 20 ng/mL	0.47 fg/mL	Gao et al., 2019
	EC	AuNPs	1–1100 pM	0.18 pM	Lopa et al., 2019
	EC	Carbon nanotubes	NA	0.05 ng/mL	Vasantham et al., 2020
	EC	Nanospheres	0.005 ng/mL to 100 ng/mL	5 pg/mL	Singh et al., 2019
	EC	Carbon nanotubes	0.05 ng/mL to 30 ng/mL	0.03 ng/mL	Shen et al., 2019
	EC	Nanorods	0.01 ng/mL to 10 ng/mL	NA	Sandil et al., 2019
	EC	Nanocubes	NA	33.3 fg/mL	Zhang et al., 2019
	EC	Nanoparticles	1.0 pg/mL to 100 ng/mL	0.39 pg/mL	Ma et al., 2019
	EC	Nanocubes	100 fg/mL to 250 ng/mL	33 fg/mL	Lv et al., 2019
	SERS	AuNPs	0.01 ng/mL to 1000 ng/mL	5 pg/mL	Fu et al., 2019
	SPR	AuNPs	NA	3.75 ng/mL	Chen et al., 2019
	FET	Nanoribbon	NA	1 pg/mL	Liu Q. et al., 2016
	ELISA	Nanodendrites	0.1 pg/mL to 1000 ng/mL	0.056 pg/mL	Jiao et al., 2019
	LFA	AuNPs	NA	0.1 ng/mL	Lim et al., 2019
	LFA	Nanosphere	0.049 ng/mL to 50 ng/mL	0.049 ng/mL	Lou et al., 2019a
	LFA	Nanosphere		0.1 ng/mL	Lou et al., 2019b
NT-proBNP	ECL	Nanocube and AuNPs	0.25 pg/mL to 100 ng/mL	0.11 pg/mL	Dong et al., 2019
MB	ECL	AuNPs and platinum nanowires	3.0 ng/mL to 0.32 μg/mL	0.11 ng/mL	Zou et al., 2019
	Fluorescence	UCNPs	10 ng/mL to 400 ng/mL	0.21 ng/mL	Ji et al., 2019
	EC	AuNPs and nanosheets	0.1 μg/mL to 100 μg/mL	34.6 ng/mL	Adeel et al., 2019
cTnT	EC	Carbon nanotubes	0.1 pg/mL to 8.0 pg/mL	0.04 pg/mL	Phonklam et al., 2020
BNP	FET	Nanoribbon		10 pg/mL	Liu Q. et al., 2016
FABP	Fluorescence	Nanowires	2.5–60 ng/mL	1.36 ng/mL	Guo et al., 2019
CK-MB	ECL	AuNPs, Fe_3_O_4_, carbon nano-onions	10 ng/mL to 50 fg/mL	5 fg/mL	Adhikari et al., 2019
	SERS	AuNPs	NA	9.7 pg/mL	Cheng et al., 2019
	FET	Nanoribbon	NA	0.1 ng/mL	Liu Q. et al., 2016
	LFA	AuNPs	NA	5 ng/mL	Lim et al., 2019
Vaspin	Fluorescence	UCNPs	0.1 ng/mL to 55 ng/mL	39 pg/mL	Ali et al., 2020
GPBB	LFA	AuNPs	NA	10 ng/mL	Lim et al., 2019

### Electrochemiluminescence (ECL) Immunoassay

Electrochemiluminescence (ECL) involves electron-transfer reactions that generate excited states and light emission, thus works as a common diagnostic assay to quantify expression levels of biomarkers because detected emission intensity in ECL is proportional to the concentration of the biomarkers (Richter, 2004). Luminol is one of the most important signal enhancers in ECL but has a weak signal and poor solubility. Fortunately, luminol-functionalized nanomaterials can overcome the limitations of traditional luminol and greatly enhance the intensity and sensitivity due to their large specific surface area (e.g., carbon nanotubes, nanosheets), the ability to functionalize signal amplification materials (e.g., nucleic acid isothermal amplification, HCR signal amplification), conductivity, and surface charge. All of these advantages have contributed to intense attention of ECL assay in biomolecule analysis (Hao et al., 2014; Feng et al., 2016).

**FIGURE 2:**
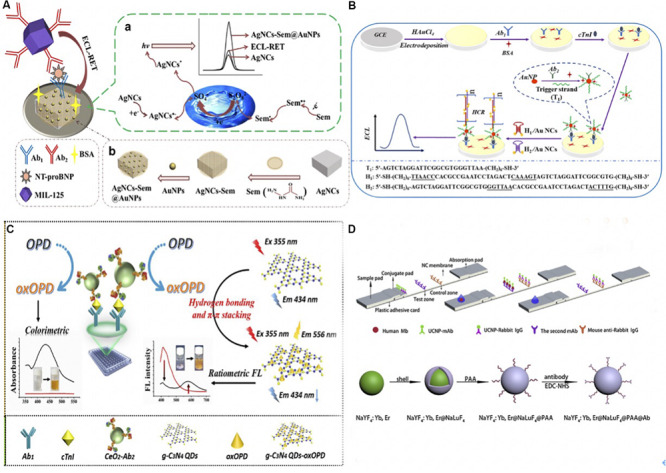
**(A) (a)** The schematic of fabricating ECL immunoassay and its possible self-enhanced luminescence mechanism. A sandwich detection format was utilized to detect NT-proBNP. Ab_1_ was immobilized via Au-NH_2_ bond and dried on AgNC-sem@AuNP modified GCE surface, once the NT-proBNP was captured, the MIL-125 labeled Ab_2_ was added to quench the ECL luminophore. The resonance energy transfer was due to the partial overlap between the ECL emission of the AgNC-sem@AuNPs (wavelength 470–900 nm) and the visible adsorption spectrum of MIL-135 (wavelength 406–900 nm). **(b)** The preparation of self-enhanced luminophore AgNC-Sem@AuNPs. AuNPs combined with Sem-AgNCs with Au-NH_2_ bond. (Dong et al., 2019) Copyright 2020 Springer Nature Switzerland AG. **(B)** Schematic Illustration of ECL-HCR Immunosensor. AuNPs modified GCE immobilized Ab_1_ via Au-N and Au-S bonds, and then AuNPs/GCE was prepared by electrodepositing in HAuCl_4_ solution. The Ab_2_-AuNP-T_1_ opened hairpin DNA structures (H1 and H2 after cTnI was caught), which triggered hybridization that modified Au NCs on the electrode. Au NCs reacted with K_2_S_2_O_2_ thus emitted the ECL signal (Zhu et al., 2019). Copyright 2020 American Chemical Society. **(C).** The mechanism of nanozyme-linked immunosorbent assay for dual colorimetric and ratiometric fluorescent detection. OPD was oxidized and converted into oxOPD by the robust nanozyme of nanoceria with peroxidase-like properties, resulting an emission maximum of oxOPD at 578 nm. OxOPD was immobilized on the surface of g-C_3_N_4_ QDs, which led to a ratiometric fluorescence response as a result of photoinduced electron transfer (PET). The combination of ratiometric fluorescent assay with colorimetric assay could quantify cTnI (Miao et al., 2019). Copyright 2020 from Elsevier B.V. **(D)** Schematic of UCNPs based immunoassay and the synthesis, surface modification of core-shell UCNPs. UCNPs-Ab_1_ and UCNPs-rabbit IgG were added on the conjugate pad, the second anti-(MB Ab_1_) and anti- (rabbit IgG) antibodies were separately stripped onto the NC membrane. Samples will be captured on the sample pad. UCNPs on the test and control line was excited by a continuous wave laser diode at 908 nm for reading. The Poly (acrylic acid) PAA was added to the core-shell NaYF_4__:_ Yb, Er@NaLuF_4_ nanoparticles, and yield UCNPs@PAA particles. Then UCNPs@PAA were conjugated with Ab_1_ using 1-Ethyl-3-(3-dimethyllaminopropyl) carbodiimide hydrochloride (EDC-HCl) and *N*-Hydroxysulfosuccinimide sodium salt (Sulfo-NHS) as cross-linking agents (Ji et al., 2019). Copyright 2020 from Elsevier B.V.

Dong et al. (2019) detected NT-proBNP using ECL immunoassay based on ECL resonance energy transfer (RET). The ECL-RET transfer was between semicarbazide-modified gold nanoparticles (AgNC-sem@AuNPs) covered nanocubes (donor) and a Ti (IV)-based metal-organic framework of type MIL-125 (receptor). The partial overlap between the ECL emission of the AgNC-sem@AuNPs and the visible adsorption spectrum of MIL-135 created RET. The schematic was illustrated in Figure 2A. Firstly, 8 μL of AgNC-sem@AuNP solution was dropped on the pre-cleaned glassy carbon electrode (GCE) surface, then the primary antibody_1_ (Ab_1_) was immobilized on it via Au-NH_2_ bond. Then, NT-proBNP analytes were added to the assay and captured by Ab_1_. Finally, the MIL-125 labeled secondary antibody_2_ (Ab_2_) was incubated onto the electrode to form the sandwich format and quench the ECL strength of luminophore. The assay was able to recover 96.8%∼100.2% of NT-proBNP and detect it as low as 0.11 pg/mL.

Zhu et al. (2019) detected cTnI using Au Nanocluster and HCR signal amplification. A sandwich immunocomplex composed of cTnI, Ab_1_, and Ab_2_-AuNP-T_1_ was applied. Ab_2_-AuNP-T_1_ is a smart probe in which the DNA initiator strands (T_1_) and Ab_2_ are conjugated onto the AuNPs. The schematic was illustrated in Figure 2B. Once the cTnI was caught, the initiator strands T_1_ of Ab_2_-AuNP-T_1_ opened the hairpin DNA structures (H1 and H2) that were dual-labeled on the Au nanoclusters (Au NCs). This process triggered hybridization events, thus modified a large number of Au NCs on the surface of the electrode. Finally, a strong ECL signal was emitted owing to the reaction of the modified Au NCs and the coreactant K_2_S_2_O_2_. This ECL-HCR sensor was able to detect 1.01 fg/mL cTnI with high specificity stability and reproducibility.

Recently, various research groups have made great progress in CVD diagnosis using ECL assay. Wang et al. (2019) combined Co^2+^-based metal organic frameworks (MOF), zeolitic imidazolate frameworks (ZIF-67), and luminol-capped Ag nanoparticles (luminol-AgNPs) for fast and ultrasensitive detection of cTnI, with a 0.58 fg/mL detection limit. Zou et al. (2019) developed an ECL immunosensor to detect MB in human serum. They fabricated a basal electrode using AuNPs and platinum nanowires that were deposited onto indium tin oxide-coated glass linked with 3-aminopropyl-trimethoxysilane. Besides, Adhikari et al. (2019) presented an ultrasensitive label-free ECL immunosensor for CK-MB detection using a novel nanocomposite-modified printed electrode. To fabricate this sensor, carbon nano-onions (CNOs)/Fe_3_O_4_/AuNP/chitosan (CS) nanocomposite was dropped in single-layered rolled-up carbon nanotubes (SWCNTs). Then Ab_1_ against CK-MB was spiked onto the electrode and blocked by bovine serum albumin (BSA). Once CK-MB was captured on the electrode, the ECL signal was determined by Tris(2,2′-bipyridyl)-ruthenium(II) chloride ([Ru(bpy)_3_]^2+^Cl) and tri-*n*-propylamine (TPrA), in which Ru(bpy)_3_]^2+^Cl was used as luminophore and TPrA was the co-reactant. Moreover, Lakshmanakumar et al. (2019) functionalized graphene quantum dots with acetic acid (fGQDs) on the Au electrode to detect cTnI. The cTnI was recognized via carbodiimide conjugation between the N-H group of cTnI and the COOH group on fGQDs instead of antibody-antigen interaction. The interaction of cTnI and fGQDs was examined by cyclic voltammetry (CV) and amperometry (Lakshmanakumar et al., 2019). They detected cTnI over a linear range of 0.17–3 ng/mL and offered a detection limit of 0.02 ng/mL with good stability and sensitivity. Based on the aforementioned discussion, ECL immunoassay is appealing for diagnosis since its high stability as well as sensitivity and specificity. Despite the considerable advantages for biomedical analysis, ECL often requires specialized and expensive equipment for generating excited states with light-emitting for detection, which to some extent impair its extensive application.

### Fluorescence Immunoassay

Fluorescence immunoassay is by far the dominant analytical approach in biomedical engineering on account of its outstanding versatility and signal enhancement ability (Strianese et al., 2012). Nanomaterials have promoted the efficiency of fluorescence assay because nanomaterials have good solubility, low toxicity, and high binding affinity of biomolecules, which can couple with various intensive fluorescence materials for amplification (e.g., Horseradish Peroxidase) (Yeh et al., 2010; Lu et al., 2017).

Miao et al. (2019) used a robust nanoceria-linked immunosorbent assay to detect cTnI based on colorimetric and ratiometric fluorescence. Ratiometric fluorescence overcomes some dependence defects, such as external environment luminophore, consequently, it can be used for trace detection. The schematic of the sensor was illustrated in Figure 2C. After the bonding of cTnI and Ab_1_, *o*-phenylenediamine (OPD) as an organic substrate was oxidized and converted into 2,3-diaminophenazine (oxOPD) by the robust nanozyme of nanoceria with peroxidase-like properties and the addition of H_2_O_2_. OxOPD was immobilized on the surface of g-C_3_N_4_ QDs through hydrogen bonding and π–π stacking interactions. The *g*-C_3_N_4_ QDs led to a ratiometric fluorescence response as a result of photoinduced electron transfer (PET). Besides, a visible color change was detected through the conversion of colorless OPD to an orange oxOPD, which acted as a colorimetric fluorescence. As a result, cTnI was quantified by combining the merits of the ratiometric assay and colorimetric assay. Similarly, Tan et al. (2019) oxidized the OPD to oxOPD through the Pd-Ir nanocubes catalysis, which possessed excellent peroxidase-like activity. The detection range of cTnI was between 1 pg/mL to 1 ng/mL, and the detection limit was 0.31 pg/mL.

Moreover, Guo et al. (2019) completed multiplexed detection of cTnI, human heart-type fatty acid binding protein (FABP), and MB using Zinc Oxide Nanowires to enhance fluorescence signal. Han and Kim (2019) designed a AuNP-Antibody-HRP conjugate for cTnI detection. They bound aldehydeactivated(ald)HRP and the primary amine group based on adsorption or covalent coupling, to enhance the sensitivity of the AuNP-based conjugates.

Upconversion nanoparticles (UCNPs) are another type of fluorescent nanomaterials that have been used in luminescent and fluorescent detection (Tan et al., 2016). Ji et al. (2019) used core-shell upconversion nanoparticles (UCNP) to capture the MB in blood samples. MB was fixed between UCNPs-Ab_1_ and second anti-(MB Ab_1_) antibody to form a sandwich format. UCNPs were excited by a continuous wave laser diode at 908 nm for fluorescent reading by external equipment. The detailed procedure was illustrated in Figure 2D. Applied UCNPs increased the sensitivity of the assay, which reached a limit of detection as low as 0.21 ng/mL with a 90.6–110.5% MB recovery rate (Ji et al., 2019). Ali et al. (2020) also designed a biosensor assisted with UCNPs for ultrasensitive detection of Vaspin.

### Photoelectrochemical (PEC)

The photoelectrochemical (PEC) process refers to the electricity conversion of photons resulting from photoactive materials absorbing photons upon illumination and forming electron-hole pairs. This, in turn, causes the oxidization-reduction reaction of the molecules and generates charge separation and subsequent charge transfer. PEC is a promising diagnostic tool since the detected photocurrent change is caused by the biological interactions between biomarkers and corresponding recognitions (Zhao et al., 2014). Nanomaterials have improved the sensitivity of PEC assay because they present a low background signal. For instance, AuNPs, QDs, TiO_2_ nanotubes (NTs), Pt NPs, and Silica NPs are suitable candidates for PEC biosensors thanks to their efficient photocurrent quenching ability and unique plasmonic properties (Zhao et al., 2012).

**FIGURE 3:**
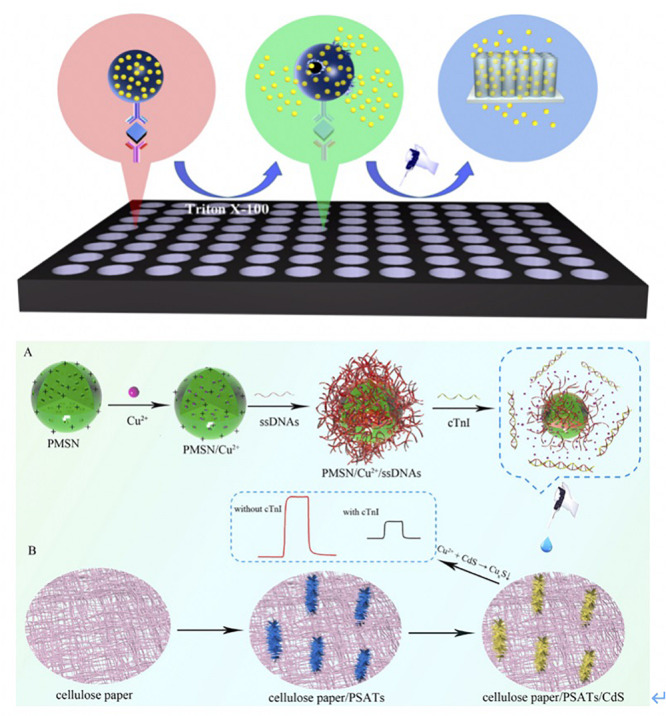
**(A)** Scheme of the proposed liposomal PEC Immunoassay. Ab_1_ were first loaded into 96-well plates, and then samples were incubated with Ab_1_ for 1 h. The QDLL-Ab_2_ was introduced after the immunorecognition of cTnI. Triton X-100 (10%) was introduced to release the QDs that were later captured by TiO_2_-NTs electrode. The photocurrents were generated due to the sensitization effect (Xue et al., 2019). Copyright 2020 American Chemical Society. **(B)** Schematic principle of cTnI detection using PMSN/Cu2+/ssDNAs. Positive-charged PMSNs were added into Cu^2+^ solutions. Cu^2+^ entered the pores of PMSNs via diffusion. Later, ssDNAs bound cTnI specifically was added and attached on the surface of PMSNs. Once the cTnI was recognized by ssDNAs, the complexes reacted with CdS that was functionalized onto the PSATs, to form Cu_*x*_S and diseased the photocurrent signal. The changes of photocurrent were correlated to the concentration of cTnI (Gao et al., 2019). Copyright 2020 from Elsevier B.V.

Xue et al. (2019) reported a split-type liposomal PEC immunoassay composed of immunoaffinity, cadmium sulfide (CdS) quantum dots-loaded liposomes (QDLL), and TiO_2_ nanotubes (NTs) that capture QDs for cTnI detection. The thioglycolic acid (TGA)-capped QDs and QDLL were linked with the Ab_2_ as signaling probes. The schema was explained in Figure 3A. cTnI was captured by Ab_1_ in the 96-well plates, and then QDLL-Ab_2_ was introduced after the immunorecognition. Triton X-100 (10%) was introduced to damage the QDLL and release the QDs that were later captured by the TiO_2_-NTs electrode. TGA-capped CdS QDs reacted with the Titania surface through the complexing between the carboxylic acid functionality on the CdS QDs and the hydroxyl groups of the TiO_2_ NTs – via either chelating or bidentate binding modes (or both). Owing to the sensitization effect, the photocurrents were acquired for PEC immunoassay.

Similarly, Dong et al. (2020) modified the indium tin oxide-polyethylene terephthalate (ITO-PET) electrode with Bi_2_Se_3_ and the flower-like ZnIn_2_S_4_ nanospheres (ZIS). The latter was synthesized by a solvothermal method, which accelerated the electronic transition and improved the photocurrent conversion efficiency. Additionally, the cadmium selenide (CdSe) QDs, which modified Ab_2_, were able to increase the photocurrent blocked by immunoaffinity binding. This resulted in a detection limit of 0.026 ng/mL of cTnI (Dong et al., 2020).

Gao et al. (2019) fabricated cellulose paper-based, single-crystalline, three-dimensional aloe like TiO_2_ arrays (PSATs) as the electron transporting material. It was subsequently coupled with CdS to form PSATs/CdS to extend the solar spectrum response for cTnI detection (Figure 3B) (Gao et al., 2019). Single stranded DNAs (ssDNAs) that bound to cTnI specifically were coupled with positive-charged mesoporous silica nanoparticles (PMSNs). The complexes were prepared as the nanocarrier to entrap the Cu^2+^, which was regarded as a signal quencher because of its reaction with CdS to form Cu_*x*_S. After the formation of cTnI-ssDNAs complexes, Cu^2+^ formed Cu_*x*_S, which decreased the photocurrent signal. This process was used to quantify the concentration of cTnI.

Despite the accomplishments of PEC assay in CVD diagnosis, PEC assay has some disadvantages, especially the inherent drawback of photoanodes. For example, the CdS photoanodes of PEC might have low charge separation and transfer efficiency. Notably, they are unstable in water upon illumination as well.

### Electrical and Reduction

Electrochemical (EC) immunoassay has attracted considerable attention and is one of the most promising techniques for diagnosis owing to its high sensitivity and fast response time. Besides that, nanomaterials are highly appreciated for improving EC sensitivity and intensity because of their catalytic property, conductivity, binding affinity, and large surface area. Like, AuNPs can bind biomolecules and facilitate electron transfer for catalyzing electrochemical reactions (Zhang et al., 2014). Besides, nanosheets and other hollow-structured nanomaterials can increase the loading efficiency of agents on grounds of their large surface area that enhances signal (Zhu et al., 2017).

Lopa et al. (2019) developed a AuNP modified titanium (Ti) metal substrate to detect cTnI based on DNA aptamer. AuNPs were deposited on Ti sheets by the potential-step deposition method. They immobilized the DNA aptamer using a self-assembled monolayer mechanism. The sensor obtained high sensitivity and specificity with the assistance of the AuNP-Ti layer and detected cTnI with the minimum detection limit of ca. 0.18 pM (Lopa et al., 2019).

**FIGURE 4:**
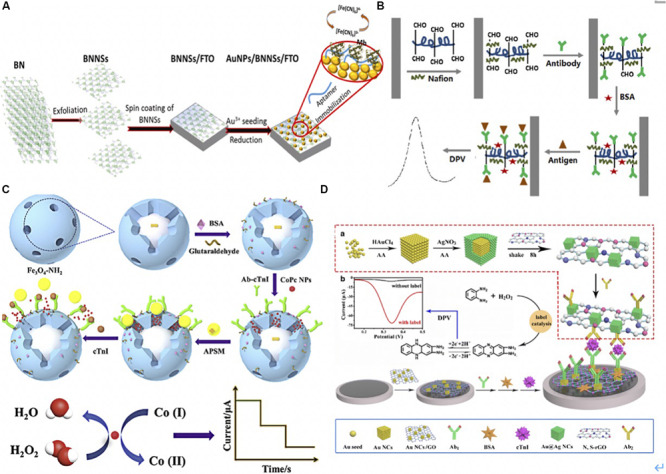
**(A)** Schematic illustration for aptasensor fabrication and MB detection process. BNNSs were obtained from filtered BN powder dissolved solution via hydrothermal method. BNNSs were spinning deposited onto FTO electrode. AuNPs were then functionalized onto BNNS/FTO electrode to form AuNPs/BNNSs/FTO electrode acted a transducer to fix a thiol-functionalized DNA aptamer (Apt) via the Au-S covalent interaction. The [Fe(CN)6]3-/4- was used as a redox probe to monitor the oxidation current variation after MB binding to the Apt (Adeel et al., 2019). Copyright (© 2020 from Elsevier B.V. **(B)**. The illustration of electrochemical immunosensor. DIL was noncovalently non-covalently bonded with HCNTs to form the DIL-HCNTs composite, which provided sufficient binding sites for Ab1. After antigen was capture by Ab1, the sandwich immunocomplexes formed on the electrode hindered electron transfer thus decreased the peak current of DPV. The signals were corresponded to concentrations of cTnI (Shen et al., 2019). Copyright 2020 American Chemical Society. **(C)** The preparation procedure of the sandwich-type electrochemical immunosensor. Dispersed Fe_3_O_4_-NH_2_ and glutaraldehyde was stirred to form Fe_3_O_4_-Ab_1_, BSA was added to block remaining active sites on the surface of Fe_3_O_4_. CoPc NPs were first dispersed in the mesoporous of Fe_3_O_4_-Ab_1_, then APSM was used to cap on the mesoporous of Fe_3_O_4_-Ab_1_ by electrostatic interaction and formed APSM-capped CoPc NPs- Fe_3_O_4_-Ab_1_. Once the target cTnI was captured, CoPC NPs were released after APSM was separated. The CoPC NPs oxidized the cobalt element from Co (I) to Co (II) with H_2_O_2_. The reduction current was corresponded to concentrations of cTnI (Ma et al., 2019). Copyright 2020 from Elsevier B.V. **(D)** The schematic diagrams of preparing Au@AgNC/N, S-rGO-Ab_2_. Au solation was mixed with AA and AgNO3 in turn, then the Au@AgNC were collected using centrifugation. Later, Au@AgNC and S-rGO were reacted for 8 h for Au@AgNC and S-rGO composite. Ab_2_ solution was added into Au@AgNC/N and S-rGO, and oscillated to form Au@AgNC/N, S-rGO-Ab_2_. AuNC/GO immobilized Ab_1_ via amino-Au affinity. Once cTnI was captured, the catalyzed-oxidation of o-phenylenediamine (o-PD) with H_2_O_2_ was accelerated. The oxidation generated 2,3-diaminophenazine that gained in electrons and hydrogen and generated a larger current signal of DPV at 0.34 V (Lv et al., 2019). Copyright 2020 Springer Nature Switzerland AG.

Moreover, Adeel et al. (2019) modified the boron nitride nanosheets (BNNS) with AuNPs to detect MB in a low-cost, label-free and simple way (Figure 4A). BNNSs were synthesized via the hydrothermal method and were deposited on the fluorine-doped tin oxide (FTO) electrode. Subsequently, AuNPs were chemically deposited on the BNNS/FTO electrode. The AuNPs/BNNSs/FTO electrode was then used as a transducer to fix a thiol-functionalized DNA aptamer (Apt) via the Au-S covalent. When the MB bound to the sensor, [Fe(CN)6]3-/4- was used as a redox probe to monitor the oxidation current variation. The Apt/AuNPs/BNNSs/FTO sensor showed a high signal response for MB, with a detection limit of 34.6 ng/mL.

Phonklam et al. (2020) presented a molecularly imprinted polymer-based (MIP) EC sensor using a screen-printed carbon electrode (SPCE) to detect cTnT. The MIP sensor possessed an electrodeposited polymethylene blue (PMB) redox probe on SPCE, which was functionalized with multi-walled carbon nanotubes (MWCNTs). Also, the electropolymerized polyaniline was around the cTnT immobilized platform. The sensor response was stimulated by pulse voltammetry, wherein the concentration of cTnT was negatively associated with the PMB current. The linearity range of the sensor was between 0.10–8.0 pg/mL, with a detection limit of 0.040 pg/mL. Similarly, Vasantham et al. (2020) integrated MWCNT with Ab_2_ to detect cTnI via paper-based multi-frequency impedimetric transducers. The limit of detection was 0.05 ng/mL, and the response time was ∼1 min.

Singh et al. (2019) developed a microfluidic biosensor-integrated mesoporous nickel vanadate hollow-nanosphere modified chitosan (Ch-Ni_3_V_2_O_8_) to detect cTnI in patient samples. They synthesized chitosan-based (Ch) Ni_3_V_2_O_8_ hollow-nanospheres that could load abundant antibodies made possible by their hydroxy and amino clusters in the nanocomposite, their good adhesion capability, and their larger surface. Moreover, they amplified the electrochemical readouts because of tunable oxidation states of the Ch-Ni_3_V_2_O_8_ matrix. The microfluidic biosensor was fabricated using a three-electrode system, including a patterned gold (Au), silver (Ag/AgCl), and Ch-Ni_3_V_2_O_8_ electrodes on a glass substrate. A bare Au electrode represented as the counter electrode (CE). The Ch-Ni_3_V_2_O_8_ composite was added on the Au-coated substrate to capture cTnI antibodies and connect the microchannels. Ag/AgCl was deposited on the substrate using e-beam evaporation for the reference electrode (RE). This device offered 5 pg/mL limit detection of cTnI with high stability, good selectivity, and high reproducibility, and detected BNP, MB, cardiac troponin C (cTnC), and cTnT with specific antigens (Singh et al., 2019).

Shen et al. (2019) presented a label-free electrochemical immunosensor using a helical carbon nanotube (HCNTs)-supported aldehyde-functionalized ionic liquid (DIL). The DIL-HCNTs provided binding sites for Ab_1_, which simplified the sensor construction processes (Figure 4B) (Shen et al., 2019). The sandwich immunocomplexes influenced electron transfer on the electrode, thereby decreasing the peak current of differential pulse voltammetry (DPV). The signals corresponded to concentrations of cTnI.

Additionally, Sandil et al. (2019) immobilized cTnI (Ab_1_) on tungsten trioxide nanorods (WO_3_ NRs), which were deposited on indium tin oxide (ITO) using the electrophoretic deposition technique that worked as the electrode. The authors detected cTnI in a linear detection range of 0.01–10 ng/mL with high reproducibility (Sandil et al., 2019). Zhang et al. (2019) synthesized β-cyclodextrins-functionalized (CDs) 3D porous graphene-supported Pd@Au nanocubes (NCs) for cTnI detection. CDs were able to increase the dispersibility of the 3D-porous graphene and improve the capability of Ab_2_ capture. Besides, the electrochemical signal was improving given the Pd@Au NCs. In addition, the amino-functionalized microporous carbon sphere was functionalized using AuNPs and Th (AuNPs-FMCS-Th) to immobilize Ab_1_ effectively and accelerate the electron transfer process. The transfer was based on the reduction of H_2_O_2_ on the Th-modified electrode surface, which further amplified the signal response. The authors detected cTnI with a low detection limit of 33.3 fg/mL through the EC signal amplification labeling in the sandwich format (Zhang et al., 2019).

Similarly, Ma et al. (2019) utilized mesoporous Fe_3_O_4_-NH_2_ for loading cobalt phthalocyanine NPs (CoPc NPs) and captured Ab_2_ to form Fe_3_O_4_-Ab_1_ to detect cTnI. The aminated polystyrene microsphere (APSM) was used to cap on the mesoporous of Fe_3_O_4_-Ab_1_ by electrostatic interaction and presented as a molecular gate. Once the target cTnI was captured, CoPC NPs were released after APSM was separated. The CoPC NPs showed a superb catalytic performance that oxidized the cobalt element from Co (I) to Co (II) with the addition of H_2_O_2_. The reduction of current corresponded to concentrations of cTnI. This novel controlled release system-based EC immunoassay presented a broad linear range from 1.0 pg/mL to 100 ng/mL with a low detection limit of 0.39 pg/mL (Figure 4C) (Ma et al., 2019). Lv et al. (2019) functionalized the gold nanocube with graphene oxide (AuNC/GO) as the substrate to immobilize Ab_1_ via amino-Au affinity. To detect cTnI, the authors utilized Au@Ag core–shell nanocubes and nitrogen/sulfur doped GO as signal amplification labels to conjugate with Ab_2_ via Ag-N bonds. Once cTnI was captured, the catalyzed-oxidation of o-phenylenediamine (o-PD) with H_2_O_2_ was accelerated. The oxidation generated 2,3-diaminophenazine that gained in electrons and hydrogen and released an exaggerated current signal of DPV, which was used to electrochemical detect cTnI (Figure 4D) (Lv et al., 2019).

EC assay has shown advances in the sensitivity, but disadvantages remain. More specifically, the facility requires periodical calibration and care maintenance to guarantee accuracy, which is expensive and bothersome.

### Surface-Enhanced Raman Scattering (SERS)

Surface-enhanced Raman scattering (SERS) immunoassay shows strong potential for CVD clinical diagnosis in view of their excellent multiplexing ability, high sensitivity, and large dynamic range (Huang et al., 2019). Typical SERS-based immunoassay uses substrates modified with Ab_1_ to capture targets, and the concentration of the targets is quantified by the SERS immunoprobe (Wang et al., 2017c). Nanomaterials have simplified the preparation and attachment of Raman labels thus have improved the sensitivity. For example, Fu et al. (2019) developed a capture probe/target/SERS nanotags platform to detect cTnI. They functionalized graphene oxide (GO) with AuNPs, which acted as both SERS nanotags and signal amplification carriers. The GO/AuNP complexes provided strong SERS enhancement ability and detected cTnI with a 5 pg/mL detection limit (Fu et al., 2019). Similarly, Cheng et al. (2019) loaded targets and polyclonal-antibody-conjugated Au@Ag core–shell nanoparticles in a gold-patterned chip that ran as a SERS active template for the ultrasensitive detection of cTnI and CK-MB. The limits of detection were 8.9 pg/mL and 9.7 pg/mL for cTnI and CK-MB, respectively.

While large surface area and outstanding physical properties of nanomaterials have facilitated sensitive SERS immunoassays, additional work is required on stabilizing the tag functionalization in SERS assays.

### Surface Plasmon Resonance (SPR)

Surface plasmon resonance (SPR) biosensor measures the changes of the refractive index at the sensor surface. Its principle is based on charge-density oscillation that exists at the interface of two media, normally involving a metal (i.e., gold) and a dielectric. SPR has been used to detect proteins, DNA, and drugs (Mullett et al., 2000). SPR sensors are mainly classified as sensors with angular wavelength and intensity modulation (Dudak and Boyaci, 2009). Nanomaterials with plasmonic and optical properties, good distributing ability, strong photostability have amplified signals, hence, improved SPR sensitivity (Liu et al., 2013).

**FIGURE 5:**
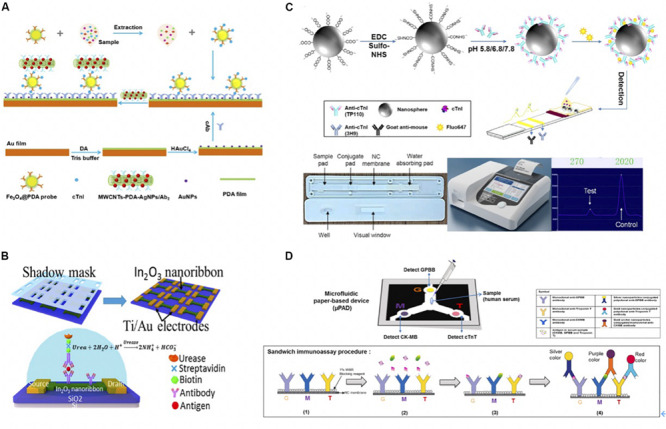
**(A)** The schematic diagram of experimental procedure. A bare Au film with soaked into DA solution in Tris-buffer to form PDA-Au film. Afterward, AuNPs were deposited onto PDA-Au film with the help of HAuCl_4_ to improve the sensitivity. After 30 min, Ab_1_ was immobilized on the PDA-Au for further cTnI capture. As for detection probe, Fe_3_O_4_@PDA-detection antibody immune probe was collected with external magnet after Fe_3_O_4_@PDA and detection antibody were mixed and shake for 24 h. Then samples with different concentrations of cTnI were incubated with Fe_3_O_4_@PDA-detection antibody, and the nanoconjugates were collected by a magnet. Once the resonant wavelength of probes was stable, MWCNTS-PDA-AgNPs/secondary antibody was added to enhance SPR response signals (Chen et al., 2019). Copyright 2020 from Elsevier B.V. **(B)** Schematic of In_2_O_3_ nanoribbon biosensor and electronic ELISA for cardiac biomarker detection. The first shadow mask was attached onto the SiO2/Si wafer, and then In_2_O_3_ ribbons were deposited using radio frequency (RF). Nanoribbons were obtained after removing first-layer shadow mask. A second shadow mask was attached to define the Ti/Au disposition, which used e-beam evaporation. Finally, a FET-based sensor was completed after removing shadow mask. Captured antibodies were fixed on the In_2_O_3_ surface and captured the biomarkers. Biomarkers were fixed between the capture antibody and a biotinylated secondary antibody that is specific to biomarkers. The biotin tales of the secondary antibody was to capture streptavidin, which bound to a biotinylated urease that led to deprotonation the hydroxyl groups on the nanoribbon and increased the negative surface charges. The change of surface charge was detected by FET sensor because negative charges decreased the conduction of nanoribbons (Liu Q. et al., 2016). Copyright 2020 American Chemical Society. **(C)** Schematic representation of the preparation of antibody-modified nanoprobes involved in the lateral flow immunoassay. Carboxylated Nanospheres (CNs) were incubated with *N*-(3-dimethylaminopropyl)-*N’*-ethylcarbodiimide hydrochloride crystalline (EDC) and *N*-hydroxysulfosuccinimide sodium salt (sulfo-NHS), the mixtures were collected centrifugation. TP110 (detection antibody) was incubated with CNs at 5.8, 6.8, or 7.8 pH. Florescent molecules were conjugated with CNs-TP110 for later detection on LFA. Briefly, sample flowed through sample pad, and conjugated with fluorescent labeled CNs-TP110 on the conjugate pad. The complexes went through NC membrane that immobilized Ab1 (3H9) on the test line and Goat anti-mouse on the control line for further antigen capture. The strip was read by a fluorescent analyzer after 15 min (Lou et al., 2019b). Copyright 2020 American Chemical Society. **(D)** Scheme of fabrication of the microfluidic paper-based device (μPAD) for multiplex detection of cardiac markers. Capture antibodies were immobilized onto NC membrane at three positions (G for GPBB, M for CK-MB, and T for troponin T). Serum sample was added at central sample zone. After antibody-antigen capture, AuNPs conjugated anti-cTnT (red), silver-nanoparticles (AgNPs) conjugated anti-GPBB (yellow) and Gold urchin nanoparticles conjugated anti-CKMB (purple) were added for color detection (Lim et al., 2019). Copyright 2020 from Elsevier B.V.

Recently, Chen et al. (2019) modified Au film with AuNPs and polydopamine (PDA), which ran as platforms for immobilizing Ab_1_ and SPR sensing. The films attracted the detection antibody on the PDA-coated Fe_3_O_4_ as the immune probe to detect cTnI (with a detection limit of 3.75 ng/mL) (Chen et al., 2019). The authors also introduced secondary antibody conjugated with multi-walled carbon nanotube (MWCNTs)-PDA-AgNPs to interact with cTnI exposed on the surface of probes, thus further amplifying the SPR response signal (Figure 5A).

### Field Effect Transistor (FET)

Field effect transistor-based (FET) sensors have been applied in the biomedical analysis on grounds of their small size, high versatility, and low costs (Syedmoradi et al., 2019). FET-based sensors use an electric field to control the flow of current on platforms such as silicon nanowire and graphene (Kaisti, 2017).

Silicon nanoribbon fabricated biosensors are highly sensitive and uniform. However, they are expensive and complicated, requiring oxidation, photolithography, and wet etching. In contrast, semiconducting metal-oxide-based field-effect transistors (FET) biosensors show advantages, as simplicity and reliability. Photolithography-free shadow mask fabrication methods are also beneficial compared to silicon-based sensors. To give an example, Liu et al. developed a highly uniform, sensitive, and reusable In_2_O_3_ nanoribbon biosensor array using a lithography-free, scalable, and facile fabrication with high time efficiency (Figure 5B) (Liu Q. et al., 2016). The nanoribbons were formed through a first-layer shadow mask that was attached to a silicon substrate using In_2_O_3_ sputter-coating. The nanoribbons went through a metal electrode deposition process that was defined by a second-layer shadow mask. Two shadow masks were simply removed instead of being lifted off, as in the case in photolithography. Captured antibodies were fixed on the In_2_O_3_ surface using *N*-(3-dimethylaminopropyl)-*N*’-ethylcarbodiimide hydrochloride/*N*-hydroxysuccinimide (EDC/NHS) chemistry. A biotinylated secondary antibody was introduced to attach biomarkers, and its biotin tales were bounded to the streptavidin that attracted biotinylated urease later. Urea increased the pH of the solution, hence, causing the deprotonation of hydroxyl groups on the nanoribbon and lowering surface potential. Consequently, the conduction of the n-type nanoribbon FETs was decreased due to increased negative surface charges. They detected cTnI, CK-MB, and BNP down to 1 pg/mL, 0.1 ng/mL, and 10 pg/mL concentration range, respectively.

### Enzyme-Linked Immunosorbent Assay (ELISA)

Enzyme-Linked Immunosorbent Assay (ELISA) is a commercialized method that identifies the concentration of targets through the color change of antigen-antibody reactions using an enzyme-linked conjugate and enzyme substrate. Even though ELISA shows advantages in reading results, it still requires more effort to improve sensitivity and accuracy (Aydin, 2015). Unique physical properties and biocompatibility of nanomaterials have greatly improved the performance of ELISA and below lateral flow assay (LFA). Nanofibers have the potential to reduce non-specific binding and improve binding efficiency due to their large surface area as an illustration. In addition, AuNPs conjugated with functional materials, such as HRP could amplify the detection signal in the assay (Wang W. et al., 2018).

Recently, Jiao et al. (2019) completed a multimodal ELISA diagnosis based on photothermal effect and the peroxidase-mimicking property in Au@Pt nanodendrites. The cTnI target was quantified using photothermal, colorimetric, and ratiometric fluorescent signals simultaneously.

### Paper Based Lateral Flow Assay (LFA)

Lateral flow assay detects targets in a fast, simple, and cheap manner that has attracted much interests in recent years (Koczula and Gallotta, 2016). Lou et al. (2019a) functionalized the LFA with a multi-layer structure, including the BSA layer to increase biotinylation sites, a streptavidin layer loaded with a fluorescent dye, and an outermost layer on which the biotinylated antibody is bounded to the streptavidin. Due to the high loading efficiency of fluorescent molecules, the authors detected the cTnI within the range of 0.049–50 ng/mL (Lou et al., 2019a). Recently, Lou et al. (2019b) conjugated Ab_2_ onto polystyrene nanospheres at pH 5.8 for fast and sensitive immunodetection. At pH 5.8, the tail-on orientated antibodies (TP110) were conjugated on the carboxylated nanospheres (CNs) because of the charge distribution and the hydrophobic area of antibodies. As a result, the loading capacity of TP110 was increased, thus the authors detected cTnI with high sensitivity. The detailed procedure was illustrated in Figure 5C. Lim et al. (2019) utilized AuNPs conjugated anti-cTnT, silver-nanoparticles (AgNPs) conjugated anti-GPBB, and Gold urchin nanoparticles conjugated anti-CKMB as probes to detect multiplexed biomarkers simultaneously. The serum sample was loaded on the central sample pad and flowed through the NC membrane where anti-GPBB, anti-CK-MB, and anti-cTnT were immobilized on at three positions. Nanoparticles-conjugated detection antibodies were added on the NC membrane for color detection in the end. The detailed illustration was in Figure 5D (Lim et al., 2019).

Despite the convenience of ELISA and LFA in analyzing biomolecules, they have some drawbacks. ELISA suffers from complicated procedures, long assay duration (at least 2–3 h), and large sample consumptions. LFA requires labor-intensive preparations and is highly susceptible to false positives/negatives due to improper operations.

## Nanotechnology-Based MOI

Nanomaterials with good bioavailability and versatility have increased the accuracy and specificity of clinical MOI applications thanks to improved resolution, signal amplification, and simple manipulation. Among other nanomaterials, nanoparticles are suitable for MOI since their mobilities in both internal and external vascular systems, high surface area to volume ratio, and imaging functionality. These advantages allow them to circulate through human bodies with low restrictions and produce functional imaging vehicles as contrast agents when they are applied in MOI settings, which leads to significantly improved diagnosis efficiency.

Nanoparticles can be used for RNA detection in intra-vascular systems because of their mobility. Injected or swallowed nanoparticles that are functionalized with MOI detectable molecules can circulate through the human body and target specific RNA for diagnosis. However, the size, shape, morphology, and density of functionalized nanoparticles should be explored carefully since the RNA detection requires nanoparticles entering and interacting with cells.

In addition, nanoparticles can work as nanoscale contrast agents by incorporating materials such as photoacoustic, fluorescent, radioactive, paramagnetic, superparamagnetic, electron-dense, light-scattering particles, and multimodal functional groups that are detectable by MOI.

The fluorescent material is a popular choice to conjugate with nanoparticles. For example, fluorescence labeled quantum dots (QDs) exhibit good performance in atherosclerotic plagues imaging (Jaffer et al., 2007). Popular radionuclides, including ^18^F, ^64^Cu, ^68^Ga, ^124^I, ^86^Y, have been conjugated with QDs, UCNPs, AuNPs, and NCs and have demonstrated good diagnostic functions (Saraste et al., 2009). For instance, radiolabeled nanoparticles play significant roles for atherosclerotic plagues under PET (Dobrucki and Sinusas, 2010). Radionuclides can be labeled either on the surface or encapsulated inside nanoparticles.

Additionally, superparamagnetic nanoparticles, like iron oxide (IO) nanoparticles and predominately magnetite (Fe_2_O_3_/Fe_3_O_4_), can improve the sensitivity of MRI by providing dark contrast to enhance the signal. For instance, MRI can detect thrombus (Chen and Wu, 2011), and the composition of plaques, including the fibrous cap and necrotic core, macrophage content, plaque neovascularization, intraplaque hemorrhage, and mural thrombus using superparamagnetic nanoparticles as contrasts (Avelar et al., 2017).

Also, AuNPs with good optical properties are good signal enhancers for photoacoustic tomography (PAT) (Taruttis et al., 2010). For example, Pan et al. (2014) successfully detected thrombus using gold nanobeacons under PAT. AuNPs can enhance the signal of coherence tomography (OCT) based on light-scattering as well.

Moreover, AuNPs were also widely used in CT as contrast agents. Hyafil et al. (2007) identified macrophages in atherosclerotic plagues using iodinated AuNPs facilitated CT. Kim et al. (2012) used polyethylene glycol (PEG)-coated AuNPs to improve the performance of CT scanning. They utilized PEG-coated GNPs as a contrast agent, in which the PEG-coating prevented antibiofouling and extended lifetime of AuNPs in the bloodstream. The PEG-coated GNPs obtained a better X-ray absorption coefficient and thus provided high-resolution images.

## Conclusion and Future Perspective

Current clinical challenges of CVDs include generating straightforward, accurate diagnostic clinical decisions, and monitoring drug responses frequently. To overcome these difficulties, various platforms have been proposed. Among these platforms, CIAs have gained attraction since they can measure and evaluate the expression of biomarkers for the prediction and diagnosis of disease in a sensitive, rapid, cheap, and non-invasive manner. Additionally, MOI settings have emerged as promising techniques for CVD diagnosis thanks to their excellent imaging capabilities that facilitate physicians to make decisions. Even though significant advances in CIAs and MOI have been made regarding CVD diagnosis, the early-stage diagnosis is still challenging since symptoms of early-stage CVDs are vague, and the expression level of cardiac biomarkers at early-stage is relatively low for detection.

Therefore, novel platforms with improved sensitivity and specificity are required. As summarized in this review, nanotechnology has greatly contributed to the developments of MOI and CIA on account of its specific physicochemical properties. By virtue of their excellent biocompatibility, nanomaterials can conjugate with various biomolecules because their excellent biocompatibility. These functional biomolecules were able to increase the sensitivity and specificity of diagnostic platforms. For example, AuNP-conjugated capture antibody can reduce non-specific bindings, thereby increasing the capture specificity and eliminating background noises in various CIAs. Besides, fluorescence lumiphore conjugated nanomaterials can work as enhancers to amplify the detective signals in ELISA, or as contrast agents in MOI. In addition, except for functionalized with other biomolecules, nanomaterial itself can serve as a signal enhancer. This is made possible by their unique optical, electrical and plasmonic properties, silica NPs can quench photocurrent effectively in PEC biosensors as an example. Moreover, nanomaterials with a large surface area (e.g., nanocubes) can improve the loading efficiency of biomolecules to increase the sensitivity of CIAs. To better elucidate CIAs in CVD diagnosis, the lasted outcomes of nanotechnology assisted CIAs (e.g., ECL) was introduced, with comparison to the sensitivity and detection range of cardiac targets among these assays. We also reviewed the clinical applications of nanoparticles in MOI settings. In this regard, we believe strongly bridging clinical platforms and nanotechnology is necessary for directing future research plans.

In this review, we have highlighted the advantages of CIAs. They are non-invasive, cheap, sensitivity, and convenient. Its repeat-sampling ability also makes it ideal for long-term diagnosis and prognosis. However, the CIA testing still faces challenges. An important concern is the sensitivity and specificity of the biomarker. Previous researchers have shown that a single marker may be insufficient, lack sensitivity and specificity for accurate CVD diagnosis. It is unrealistic to provide clear diagnosis results using only one biomarker owing to the complexity, heterogeneity and diversity of pathogenesis in different populations. Additionally, biomarkers may be regulated differently during the development of pathologies. Consequently, even though we can diagnosis some clinical subjects who have high risks for certain diseases using biomarkers (e.g., cTnI for AMI), we have insufficient information on the progression and states of the diseases (Madhurantakam et al., 2018). To solve this problem, multiplexed biomarker panels are critical. The multiplexed analysis of various biomarkers can establish correlations (or scores) with high specificities and predictive values, as a result, can improve the accuracy of diagnostic decisions. Hence, the fabrication of CIAs should focus on multiplexed analysis in the future. In addition, current findings on biomarkers are based on relatively small samples, which may possess large variations (Sayed et al., 2014). Most of the studies only focused on the correlation between biomarker expression level and single disease. The pathological significance behind the biomarker expression level changing is not addressed yet. As such, large-scale and systematic clinical studies are needed to recognize and better understand the mechanisms of biomarkers. Most importantly, combination with advanced analytic tools (e.g., machine learning) that is capable of developing objective and automatic algorithms to analyze large-scale and high-dimensional-multiplexed data should be highlighted in future studies, which are supposed to greatly improve the efficiency and accuracy of diagnosis.

## Author Contributions

ZY and JZ coordinated this project. CS and HX wrote this manuscript. CS and HX collected and summarized the literatures. YM edited the figures in this manuscript and revised this manuscript.

## Conflict of Interest

The authors declare that the research was conducted in the absence of any commercial or financial relationships that could be construed as a potential conflict of interest.

The reviewer MK declared a shared affiliation, with no collaboration, with one of the authors, YM, to the handling Editor at the time of review.
